# Impact of Hidradenitis Suppurativa on Sexual Quality of Life

**DOI:** 10.3390/jcm14030910

**Published:** 2025-01-30

**Authors:** Annik Caliezi, Andrea Rabufetti, Robert Hunger, Ronald Wolf, S. Morteza Seyed Jafari

**Affiliations:** Department of Dermatology, University Hospital of Bern, 3010 Bern, Switzerlandronald.wolf@insel.ch (R.W.)

**Keywords:** acne inversa, hidradenitis suppurativa, sexual distress, sexual dysfunction, sexual quality of life, relationship

## Abstract

Hidradenitis suppurativa (HS) is a chronic inflammatory skin condition that affects about 1% of the world’s population. It is characterized by round, painful nodules, abscesses or sinuses, often in the genital area. HS has the worst impact on quality of life (QoL) of any dermatological condition. **Methods**: The aim of this systematic review is to analyze how HS affects patients’ sexual quality of life (SQoL), herein defined as a person’s evaluation of their sexual relationships, including physical and mental aspects, and their response to this evaluation. **Results**: The systematic search yielded 41 primary results. After screening, 6 studies were selected for this review. Men with HS suffer from lower SQoL than male controls and sexual dysfunction is more common in both male and female patients than in controls. Sexual dysfunction is worse in all HS patients than in controls, and worse in female patients than in male patients. Disease severity is not related to any of the sexual concepts analysed. **Conclusions**: HS has a strong impact on SQoL, as patients suffer from sexual dysfunction and sexual distress more often than healthy controls, and feel that their relationships are negatively affected by the disease. Therefore, the impact of HS on SQoL should be further investigated, especially the psychological aspect of SQoL.

## 1. Introduction

Hidradenitis suppurativa (HS) is a chronic inflammatory skin disease that affects approximately 1% of the population and is more common in women and younger people [1,2,3,4]. Lesions begin as round, painful nodules accompanied by symptoms of pruritus, hyperhidrosis and warmth [4]. The nodules may develop into abscesses and sinus tracts, which may rupture, releasing foul-smelling discharge [4]. Due to the chronic nature of the disease, scarring, fibrosis, fistulas, lymphedema and squamous cell carcinoma are complications of HS [1,3,4]. Lesions are typically found in the axillary, mammary and genital regions [1,3,5]. The etiology of HS is complex and multifaceted. Follicular alterations lead to follicular occlusion, rupture into the dermis and an inflammatory response [3,6,7]. Risk factors for HS include nicotine use, obesity and genetic predisposition [3,4,6,7]. HS can be treated conservatively or surgically, but there is no cure [2,8,9].

It is widely accepted that the quality of life (QoL) of almost all HS patients is affected by the disease, more so than most other skin diseases [10,11,12,13,14,15]. This impairment of QoL is due to many different factors, both physical and psychological. Symptoms can interfere with everyday tasks, such as dressing, leaving patients with feelings of helplessness and thus impairing QoL [1,10,12]. In addition, patients struggle with poor mental health due to low self-esteem, depression, social isolation, fear of social rejection, and more [1,12,13,16]. This physical and psychological burden on HS patients often leads to their inability to work and thus to financial problems [10,12,13].

Many chronic diseases and/or their medications can affect sexual quality of life (SQoL) [17] (Table 1). There are many questionnaires to measure the SQoL, but most studies have used the SQoLM for SQoL in men, the FSDS-R for sexual distress in women, and the FSFI and IIEF for sexual dysfunction in women and men, respectively [18,19,20]. The equivalent of the SQoLM for women, the SQoLF, also exists but is less commonly used in dermatological research. In addition, item 9 of the DLQI is often used to measure the sexual aspect of quality of life [21,22]. In dermatological diseases, such as psoriasis, the same scores are typically used to assess sexual parameters, namely the SQoLM, the FSFI, IIEF, FSDS-R, and the DLQI, specifically Item 9 [21,22,23,24]. The effect of HS on patients’ SQoL has only been investigated in one study, using the SQoLM and therefore only analyzing male patients [18]. Other sexual concepts, such as sexual dysfunction and sexual distress, have been investigated more frequently in HS patients and allow conclusions to be drawn about SQoL. HS has a debilitating effect on patients’ QoL, but their SQoL has not been thoroughly analyzed. This review discusses the impact of HS on SQoL.

## 2. Materials and Methods

**Figure 1:**
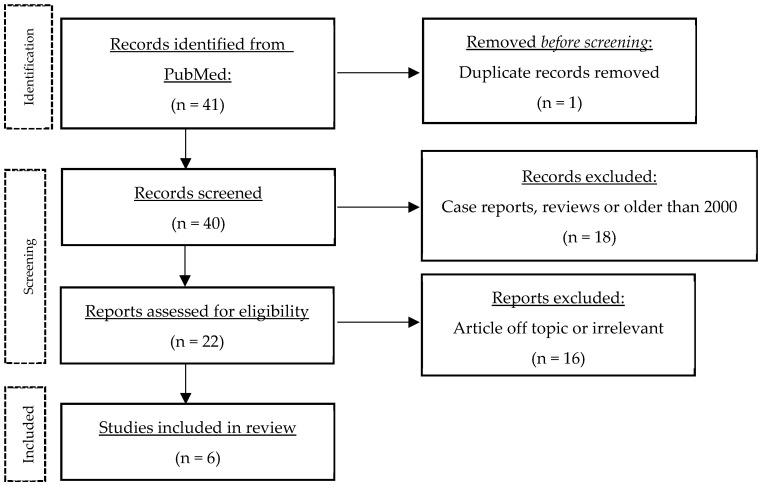
Process of literature search.

A literature search was performed on PubMed (11 July 2023) using the following keywords and variations thereof: “hidradenitis suppurativa”, “acne inversa”, “Verneuil’s disease”, “erectile dysfunction”, “sexual dysfunction”, “sexuality”, “sexual quality of life”, “sexual health”, and “sexual distress” (complete search string in the Appendix A). This search yielded 41 publications. The results were then screened to exclude case reports, systematic reviews and studies published before 2000. Articles had to be accessible via PubMed or Ovid MEDLINE All, and only German, French or English articles were included. The only hit for SQoL was a single study using the Sexual Quality of Life for use in Men (SQoLM) questionnaire. Thus, topics similar to SQoL such as sexual health, sexual dysfunction, sexual distress were included. The studies were arranged chronologically to show the changes and especially the progress made over time (Table 2). Finally, the main themes that emerged from the papers were discussed in more detail. Figure 1 shows the flow of information through the different stages of the review.

## 3. Results

### 3.1. Sexual Quality of Life

SQoL is a complex concept defined as a person’s assessment of their sexual relationships, including physical and mental health aspects (e.g., sexual dysfunction and distress) and consequences of their sexual problems [23]. Similar concepts to SQoL are sexuality, which includes facets such as gender identification, gender roles, reproduction, and sexual health [30]. The latter is defined by the WHO as “a state of physical, emotional, mental and social well-being related to sexuality” [29]. SQoL is significantly worse in male HS patients compared to healthy controls (*p* < 0.0001) [18]. Worse SQoL has a negative impact on QoL [18]. No studies have directly analyzed SQoL in women with HS, only similar concepts such as sexual distress or sexual dysfunction, which will be discussed later in this paper.

### 3.2. Sexual Distress

HS patients show higher sexual distress than healthy controls (21.4 ± 5.7 vs. 27.7 ± 4.6, *p* < 0.01), as measured with the Frankfurt Self Concept Scale for Sexuality [19]. This is supported in another study, where sexual distress is higher in female patients than in female controls (*p* = 0.002), using the Female Sexual Distress Scale- Revised [18]. There is also a difference between men and women, as men with HS have less sexual distress than women with HS (*p* = 0.02, *p* < 0.05) [19,32]. In general, patients with HS have worse QoL than healthy controls (♀: *p* < 0.001; ♂: *p* < 0.001) and in case of female patients, sexual distress is associated with QoL, which is not the case for male patients [18,19].

### 3.3. Sexual Dysfunction

According to sex-specific questionnaires, 62% of women and 52% of men with HS suffer from sexual dysfunction or erectile dysfunction [20]; according to non-specific questionnaires, the percentage ranges from 42% to 60.8% [20,31]. Sexual health is significantly worse in female HS patients than in male HS patients (*p* < 0.001) [20]. Compared to male controls, male HS patients have higher rates of erectile dysfunction (*p* = 0.01, *p* = 0.019) [18,19]. For women, the results are inconclusive, as the association was statistically significant in one study (*p* = 0.01) and not significant in another (*p* = 0.075) [18,19]. Sexual dysfunction in women is associated with their QoL, but only when gender-specific questionnaires are used [18,19,20]. The results for men are conflicting, as two studies found no association between sexual dysfunction and QoL, but another did [18,19,20]. Poorer mental health is a contributing factor to sexual dysfunction (*p* = 0.003), stable relationships do not have a significant impact, and a positive family history of HS actually decreases the risk of sexual dysfunction [20,31].

### 3.4. Sexuality

Sexual activity decreased in nearly 60% of HS patients, e.g., due to decreased sexual desire, and about the same number of patients were impaired in their sexuality because of HS, e.g., due to severe skin symptoms or anxiety [20,31]. Patients’ psychological well-being is important for their sexual desire (*p* < 0.05) [31]. Self-consciousness and reluctance to talk about sexual problems are more common in HS patients than in controls (*p* < 0.0001) [33]. Seventy-one percent of patients with a stable partner report that the disease has a negative impact on their relationship, subjectively because of symptoms such as pain or suppuration [33]. Among single patients, 90.6% (♀: 94.3%, ♂: 80.8%) feel that HS has a negative impact on their chances of having a relationship or sexual relations [33]. Controls are less likely to report fear of rejection or reaction from their sexual partner than patients with HS (37.6% vs. 47.9%, *p* < 0.05) [33].

### 3.5. Clinical HS Characteristics

Disease severity has no significant effect on sexual distress, sexual dysfunction, sexual desire, or sexual impairment [19,20,31,32]. There is a significant connection between disease severity and QoL though [20,31] The location of the lesion is important for sexual distress, as the latter correlates with lesions in the groin or on the genitals (*p* = 0. 015, *p* = 0.033) [32]. In addition, sexual distress and sexual dysfunction are worse in men with HS than in controls, but male patients without lower abdominal lesions (n = 2) have similar scores to healthy controls on these sexual measures [18,19]. This may suggest that lesion location influences sexual distress and dysfunction. However, the association with sexual dysfunction is controversial, as two other studies found no significant correlation between genital or anogenital lesions and sexual dysfunction [18,20]. Active disease is perceived by patients as having a negative impact on their relationships [33]. It also leads to poorer QoL in all patients and worse sexual dysfunction in female patients (*p* < 0.001, *p* = 0.009) [20].

## 4. Discussion

HS is a chronic inflammatory skin disease, which drastically reduces patients’ QoL [10,11,12]. Men with HS have worse SQoL than men without HS, which was also found by Yee et al. [18,34]. This result must be considered with care, given the small sample size of men (n = 17) [18]. Sexual distress is higher in patients with HS than in controls and higher in female patients than in male patients [18,19,32]. The latter difference may be due to different societal perceptions of women, where body image is more important and genitofemoral lesions are more common in women with HS [19,34,35].

Sexual dysfunction, as measured by gender-specific scores, is more common in HS patients than in the general population (HS: 52–62%, general population: 31–42%) [20,36], a finding also made by Seetan et al. in their review [37]. Male patients have worse sexual dysfunction than male controls [18,19]. The results for women are equivocal, as two studies measured worse results in patients, but only one study found this difference to be significant [18,19].

The majority of patients perceive HS to have a negative impact on their relationship or relationship opportunities [33], and struggle with self-consciousness and fear of rejection [33], which may be explained by dissatisfaction with body image, and consequences such as embarrassment, isolation, and depression, as noted by Seetan et al. [37].

Surprisingly, HS severity is not associated with sexual distress, sexual dysfunction, sexual desire or sexual impairment [19,20,31,32], but only with QoL. A comparison with psoriasis also makes these results surprising, as worse disease severity correlates with worse QoL and more sexual problems (*p* < 0.001; *p* = 0.04) [38]. Thus, this review agrees with Quinto et al. [31] who concluded that the insignificant associations between disease severity and sexual measures are controversial and that HS has a negative impact on patients’ lives, regardless of severity. In addition, sexual health in HS patients is influenced by a complex interplay of physical symptoms, psychological distress, interpersonal factors and coping mechanisms. While disease severity plays a role, its effect may be diluted by these other factors, leading to the lack of correlation observed in some studies. In addition, current HS scoring systems, such as the Hurley score, may not always accurately reflect disease activity. For example, a patient with a large, inflamed, continuously draining nodule with no sinus tracts or scarring (considered Hurley stage I) may suffer more clinically and psychologically than a patient with diffuse involvement, multiple interconnected sinus tracts and scarring, but few or no active lesions (considered Hurley stage III). This discrepancy highlights the need for more clinical/activity-oriented scoring systems (e.g., Hurley Staging Refined [39]) that better capture disease activity. Such refined tools could provide a clearer understanding of disease burden and could be used to assess QoL in general and sQoL in particular in future prospective studies. These studies should include larger sample sizes and take into account the multiple parameters affecting sQoL in HS patients, ultimately improving patient care and outcomes.

Genital and inguinal lesions are significantly associated with sexual distress [32]. On the other hand, lesion location did not affect sexual dysfunction [18,20]. Kurek et al. [19] measured these parameters in men with (n = 18) and without (n = 2) lesions of the lower abdomen. They found sexual distress and sexual dysfunction in men without lower abdominal regions to be similar to the results of healthy controls, while the average over all male patients was worse than in controls [19]. This suggests that lesion location is of importance for both sexual distress, and contrary to other findings, sexual dysfunction [18,20].

To put these results into perspective, a comparison with a more well-understood chronic skin disease like psoriasis makes sense. Most patients with psoriasis feel that the illness has a moderate to large negative impact on their QoL and alters their daily activities [40]. Like HS patients, they feel sentiments of shame and embarrassment, have a negative body image, and struggle with sexual functioning [40]. Sampogna et al. [22] and Kędra et al. [38] observed between 40% and 70% of patients to report a declined or affected sex life since the onset of disease, or as a consequence of the disease, which is similar to HS (60%) [20]. For sexual problems, the location of psoriasis lesions was significant, similar to the HS lesion location being significant for sexual distress [19,32,38]. In a direct comparison of scores between HS and genital psoriasis (Table 3), HS shows worse QoL (DLQI), worse sexual dysfunction in women (FSFI), worse sexual distress in women (FSDS-R/FSDS) and worse SQoL in men (SQoLM) [18,19,20,21,24]. Concerning erectile dysfunction (IIEF), the comparison between HS and genital psoriasis does not yield a clear result, but the trend still goes toward HS patients having worse erectile dysfunction than psoriasis patients with or without genital involvement [18,19,20,21,24]. In conclusion, HS has similar or worse effects on SQoL and sexual health than psoriasis, and lesion location matters in both diseases. Another example is atopic dermatitis (AD), which also has a significant impact on patients’ quality of life. In an interesting study of 266 patients with AD, a decrease in sexual desire due to AD was found in 57.5% of patients. The quality of life of their partners did not seem to be particularly affected, but 36.5% reported that the appearance of eczema had an impact on their sex life [41].

Limitations of this review include the small sample size of patients in each study analyzed and the different measurement tools used, which make it difficult to compare results and draw generalizable conclusions.

Future perspectives for this area of research would be to agree on which questionnaires and measurement tools to use. The concept of SQoL has only been investigated in male patients in one study, so it would be interesting to learn more about SQoL in all genders. The physical aspect of SQoL (e.g., sexual dysfunction) has been studied more thoroughly, so a better look at the mental facet of SQoL would be beneficial.

## 5. Conclusions

Overall, HS is a disease with a remarkable impact on patients’ QoL and SQoL. They struggle with problems such as sexual dysfunction, reduced sexual activity and sexual distress, which is higher in women than in men. Surprisingly, disease severity does not seem to be significant for sexual problems in HS. Patients report that HS has a negative impact on their relationships and relationship opportunities. More standardized research with more participants might be useful to work on better support and treatment for HS patients and thus improve their QoL and SQoL.

## Figures and Tables

**Table 1:** Definitions of sexual concepts.

Term	Defintion	Relation with Other Concepts
Sexual quality of life (SQoL)	A person’s evaluation of the positive and negative aspects of their sexual relationships and their response to this evaluation. It includes a person’s mental health, physical health, how a person perceives their own sexual function, and the consequences of sexual problems [23].	Includes: Sexual distress, sexual dysfunction, sexuality Overlaps with: Sexual health
Sexual dysfunction	This concept includes many different subtypes, given it is an umbrella term. It includes any disorders that is characterized by issues of the sexual-response cycle. The most important dysfunctions are disorders of sexual desire, disorders of sexual arousal, problems with orgasms, and pain [25,26]	
Sexual distress	A person’s anxiety, worry, and frustration regarding their sexual activities [27].	
Sexual health	A state of physical, emotional, mental and social well-being related to sexuality [28,29]	Includes: sexuality, sexual distress, sexual dysfunction Overlaps with: SQoL
Sexuality	A complex concept, consisting of gender, gender roles, gender identity, sexual orientation, eroticism, pleasure and reproduction [30].	Includes: sexual dysfunction, sexual distress

**Table 2:** Most important results.

Author	Patients (n)	Important Outcomes	Scores	Key Results
Quinto et al., 2021 [31]	77	Sexual dysfunction, sexual desire, hindered sexuality, mental status	Hurley, IHS4, Sartorius, VAS for pain, SDQ, SDI-2, GHQ-12, HADS, SF-36, Skindex-17, individual questions	- Hindered sexuality: 61.8%, sexual dysfunction 60.8% - Disease severity not associated with sexual dysfunction (SDQ), hindered sexuality or sexual desire (all *p* > 0.3) - Severe skin symptoms (Skindex-17) and high anxiety (HADS) significantly associated with elevated risk of hindered sexuality (*p* = 0.031, *p* = 0.021).
Cuenca-Barrales et al., 2020 [32]	386	Difficulties in sex life due to HS, perceived attractiveness, influence of relationship status	Hurley, PtGA, Self-designed questionnaire, NRS for symptoms	- 94.3% of female and 80.8% of male single patients feel HS has negative influence on their chances of having a relationship, 71.4% % of patients think HS negatively influences preexisting relationship - Fear of rejection or of reaction of their partner more common in patients than in controls (47.9%, resp. 37.6%; *p* < 0.05) - Perceived attractiveness of themselves significantly lower in patients than controls (*p* < 0.0001).
Cuenca-Barrales et al., 2019 [32]	386	sexual distress, associated factors	Hurley, PtGA, NRS for symptoms and sexual impact of HS, FSFI, IIEF	- Women’s sexual distress (NRS for HS impact on sex life) greater than men’s (*p* < 0.05) - Active lesions in groin (*p* = 0.015) and genitals (*p* = 0.033) significantly associated with higher sexual distress (NRS)
Alavi et al., 2018 [18]	50	QoL, Sexual/erectile dysfunction, SQoL in men, sexual distress in women	Hurley, DLQI, FSFI, IIEF, SQoLM, FSDS-R	- QoL (DLQI) significantly lower in HS patients than controls (*p* < 0.0001) - SQoL (SQoLM) and erectile dysfunction (IIEF) in men, and sexual distress in women (FSDS-R) worse in patients than controls (*p* = 0.019; *p* < 0.0001; *p* = 0.002) - Sexual dysfunction in women (FSFI) insignificantly worse than in controls (*p* = 0.075) - Worse SQoL in men (SQoLM) correlates with worse QoL (DLQI) (*p* = 0.001)
Janse et al., 2017 [20]	300	Sexual/erectile dysfunction, Quality of Life,	Hurley, PGA, VAS for pain, FSFI, IIEF, ASEX, DLQI	- Sexual dysfunction in 62% of female patients (FSFI), erectile dysfunction in 52% of male patients (IIEF) - 59.7% of patients report decline of sexual activity after onset of HS
Kurek et al., 2012 [19]	44	Sexual/erectile dysfunction, sexual distress, sexuality, QoL	Sartorius, FSFI, IIEF, FKKS SSEX, DLQI	- Higher sexual dysfunction in patients than controls (FSFI: *p* = 0.01, IIEF: *p* = 0.01). - Sexual distress (FKKS SSEX) higher in patients than controls (*p* < 0.01) and higher in female patients than in male patients (*p* = 0.02). - No associations between sexual dysfunction (FSFI, IIEF) or sexual distress (FKKS SSEX) and disease severity (Sartorius) (*p* = 0.34; *p* = 0.31; *p* = 0.25).

Abbreviations: ASEX: Arizona Sexual Experience Scale, DLQI: Dermatology Life Quality Index, FKKS SSEX: Frankfurt Self-Concept Scale for Sexuality, FSDS-R: Female Sexual Distress Scale—Revised, FSFI: Female Sexual Function Index, GHQ-12: General Health Questionnaire, HADS: Hospital Anxiety and Depression Scale, HS: Hidradenitis suppurativa, Hurley: Hurley Staging System, IHS4: International Hidradenitis Suppurativa Severity Score System, IIEF: International Index of Erectile Dysfunction, NRS: Numeric Rating Scale, PtGA/PGA: Patient’s Global Assessment, QoL: Quality of life, Sartorius: Sartorius Score, SDI-2: Sexual Desire Inventory-2, SDQ: Sexual Dysfunction Questionnaire, SF-36: Short Form Health Survey, SQoL: sexual quality of life, SQoLM: Sexual Quality of Life Questionnaire for Use in Men, VAS: Visual Analogue Scale.

**Table 3:** Comparison between Psoriasis—Genital Psoriasis—HS. * Meeuwis et al. [24] used the FSDS, not the revised FSDS-R, (Gen: psoriasis with genital Involvement, NGen: psoriasis with no genital involvement).

	DLQI	IIEF	FSFI	FSDS(-R)	SQoLM
Non-genital Psoriasis (Yi et al. [21])	6.5 (±6.6)	57.0 (±12.6)	63.6 (±22.3)	11.4 (±11.4)	52.6 (±18.8)
Genital Psoriasis (Yi et al. [21])	8.8 (±6.9)	48.5(±17.9)	53.5 (±24.7)	20.7 (±14.5)	51.4 (±16.3)
Genital Psoriasis (Meeuwis et al. [24])	8.5 (±6.5)	55.0 (±16.9)	23.6 (±8.8)	16.1 (±12.1) *	76.1 (±24.4)
HS (Alavi et al. [18])	± 18	±48	±17	±27	±31
HS (Janse et al. [20])	12.5 (±7.5)	49.7 (±20.7)	21.6 (±9.9)		
HS (Kurek et al. [19])	♀: 14.4 (±6.6) ♂: 9.6 (±6.9)	42.6 (±27.1)	22.1 (±10.2)		
Interpretation	HS > Gen > NGen (HS the worst)	HS rather < Gen < NGen (HS rather worse)	HS < Gen < NGen (HS the worst)	HS > Gen > NGen (HS the worst)	HS < Gen—NGen (HS the worst)

Abbreviations: DLQI: Dermatology Life Quality Index, FSDS: Female Sexual Distress Scale, FSDS-R: Female Sexual Distress Scale-Revised, FSFI: Female Sexual Function Index, IIEF: International Index for Erectile Dysfunction, SQoLM: Sexual Quality of Life Questionnaire for use in Men.

## Data Availability

The data supporting the results of this study are presented in the current paper.

## Appendix A. Complete Search String

(“verneuil disease”[Title/Abstract] OR “verneuil’s disease”[Title/Abstract] OR “Pyodermiafistulanssinifica”[Title/Abstract] OR (“Hidradenitis suppurativa”[MeSH Terms] OR “Hidradenitis suppurativa”[Title/Abstract] OR “acne inversa”[Title/Abstract] OR “suppurative Hidradenitis”[Title/Abstract] OR “hidradenitis suppurative”[Title/Abstract] OR “suppurative Hidradenitis”[Title/Abstract] OR “inversa acne”[Title/Abstract] OR “inverse acne”[Title/Abstract])) AND (“Erectile dysfunction”[MeSH Terms] OR “sexuality”[MeSH Terms] OR “sexual dysfunction, physiological”[MeSH Terms] OR “sexual dysfunctions, psychological”[MeSH Terms] OR (“sexual dysfunction, physiological”[MeSH Terms] AND “sexual dysfunctions, psychological”[MeSH Terms]) OR (“Sexual quality of life”[Title/Abstract] OR “sexual QoL”[Title/Abstract] OR “sexual impairment*”[Title/Abstract] OR “sexual health”[Title/Abstract] OR “quality of life sexual”[Title/Abstract] OR “sexual dysfunction*”[Title/Abstract] OR “quality of sexual life”[Title/Abstract] OR “sexual function*”[Title/Abstract] OR “sexuality”[Title/Abstract] OR “sexual distress”[Title/Abstract] OR “erectile dysfunction*”[Title/Abstract]))

## References

[1] Nguyen T.v., Damiani G., Orenstein L., Hamzavi I., Jemec G. (2021). Hidradenitis suppurativa: An update on epidemiology, phenotypes, diagnosis, pathogenesis, comorbidities and quality of life. J. Eur. Acad. Dermatol. Venereol..

[2] Scala E., Cacciapuoti S., Garzorz-Stark N., Megna M., Marasca C., Seiringer P., Volz T., Eyerich K., Fabbrocini G. (2021). Hidradenitis Suppurativa: Where We Are and Where We Are Going. Cells.

[3] Alikhan A., Lynch P.J., Eisen D.B. (2009). Hidradenitis suppurativa: A comprehensive review. J. Am. Acad. Dermatol..

[4] Revuz J. (2009). Hidradenitis suppurativa. J. Eur. Acad. Dermatol. Venereol..

[5] Wessel G.M. (2016). The milk line—Where mammary gland meets mathematics. Mol. Reprod. Dev..

[6] Diaz M.J., Aflatooni S., Abdi P., Li R., Anthony M.R., Neelam S., Farkouh C., Tran J.T., Svoboda S., Forouzandeh M. (2023). Hidradenitis Suppurativa: Molecular Etiology, Pathophysiology, and Management—A Systematic Review. Curr. Issues Mol. Biol..

[7] Wolk K., Join-Lambert O., Sabat R. (2020). Aetiology and pathogenesis of hidradenitis suppurativa. Br. J. Dermatol..

[8] van Straalen K.R., Schneider-Burrus S., Prens E.P. (2020). Current and future treatment of hidradenitis suppurativa. Br. J. Dermatol..

[9] Zouboulis C.C., Desai N., Emtestam L., Hunger R.E., Ioannides D., Juhász I., Lapins J., Matusiak L., Prens E.P., Revuz J. (2015). European S1 guideline for the treatment of hidradenitis suppurativa/acne inversa. J. Eur. Acad. Dermatol. Venereol..

[10] Matusiak Ł., Bieniek A., Szepietowski J.C. (2010). Hidradenitis suppurativa markedly decreases quality of life and professional activity. J. Am. Acad. Dermatol..

[11] Chernyshov P.V., Finlay A.Y., Tomas-Aragones L., Poot F., Sampogna F., Marron S.E., Zemskov S.V., Abeni D., Tzellos T., Szepietowski J.C. (2021). Quality of Life in Hidradenitis Suppurativa: An Update. Int. J. Environ. Res. Public Health.

[12] Kouris A., Platsidaki E., Christodoulou C., Efstathiou V., Dessinioti C., Tzanetakou V., Korkoliakou P., Zisimou C., Antoniou C., Kontochristopoulos G. (2017). Quality of Life and Psychosocial Implications in Patients with Hidradenitis Suppurativa. Dermatology.

[13] Alavi A., Anooshirvani N., Kim W.B., Coutts P., Sibbald R.G. (2015). Quality-of-Life Impairment in Patients with Hidradenitis Suppurativa: A Canadian Study. Am. J. Clin. Dermatol..

[14] Von Der Werth J.M., Jemec G.B. (2001). Morbidity in patients with hidradenitis suppurativa. Br. J. Dermatol..

[15] Wolkenstein P., Loundou A., Barrau K., Auquier P., Revuz J. (2007). Quality of life impairment in hidradenitis suppurativa: A study of 61 cases. J. Am. Acad. Dermatol..

[16] Matusiak L., Bieniek A., Szepietowski J.C. (2010). Psychophysical aspects of hidradenitis suppurativa. Acta Derm. Venereol..

[17] Manninen S.M., Polo-Kantola P., Vahlberg T., Kero K. (2022). Patients with chronic diseases: Is sexual health brought up by general practitioners during appointments? A web-based study. Maturitas.

[18] Alavi A., Farzanfar D., Rogalska T., Lowes M.A., Chavoshi S. (2018). Quality of life and sexual health in patients with hidradenitis suppurativa. Int. J. Womens Dermatol..

[19] Kurek A., Peters E.M.J., Chanwangpong A., Sabat R., Sterry W., Schneider-Burrus S. (2012). Profound disturbances of sexual health in patients with acne inversa. J. Am. Acad. Dermatol..

[20] Janse I.C., Deckers I.E., van der Maten A.D., Evers A.W.M., Boer J., van der Zee H.H., Prens E.P., Horváth B. (2017). Sexual health and quality of life are impaired in hidradenitis suppurativa: A multicentre cross-sectional study. Br. J. Dermatol..

[21] Yi O.S., Huan K.Y., Har L.C., Ali N.M., Chiang T.W. (2022). Genital Psoriasis: A Prospective, Observational, Single-Centre Study on Prevalence, Clinical Features, Risk Factors, and Its Impact on Quality of Life and Sexual Health. Indian. J. Dermatol..

[22] Sampogna F., Abeni D., Gieler U., Tomas-Aragones L., Lien L., Titeca G. (2017). Impairment of Sexual Life in 3,485 Dermatological Outpatients From a Multicentre Study in 13 European Countries. Acta Derm. Venereol..

[23] Riazi H., Madankan F., Azin S.A., Nasiri M., Montazeri A. (2021). Sexual quality of life and sexual self-efficacy among women during reproductive-menopausal transition stages and postmenopause: A comparative study. Womens Midlife Health.

[24] Meeuwis K.A.P., de Hullu J.A., van de Nieuwenhof H.P., Evers A.W.M., Massuger L.F.A.G., van de Kerkhof P.C.M. (2011). Quality of life and sexual health in patients with genital psoriasis. Br. J. Dermatol..

[25] Hatzimouratidis K., Hatzichristou D. (2007). Sexual dysfunctions: Classifications and definitions. J. Sex. Med..

[26] APA Dictionary of Psychology [Internet]. https://dictionary.apa.org/sexual-dysfunction.

[27] Stephenson K.R., Meston C.M. (2010). When are sexual difficulties distressing for women? The selective protective value of intimate relationships. J. Sex. Med..

[28] Ludwig C.M., Fernandez J.M., Hsiao J.L., Shi V.Y. (2020). The Interplay of Atopic Dermatitis and Sexual Health. Dermatitis.

[29] Wilmoth M.C. (2007). Sexuality: A Critical Component of Quality of Life in Chronic Disease. Nurs. Clin. N. Am..

[30] Sexual Health [Internet]. https://www.who.int/health-topics/sexual-health.

[31] Cuenca-Barrales C., Ruiz-Villaverde R., Molina-Leyva A. (2019). Sexual Distress in Patients with Hidradenitis Suppurativa: A Cross-Sectional Study. J. Clin. Med..

[32] Quinto R.M., Mastroeni S., Sampogna F., Fania L., Fusari R., Iani L. (2021). Sexuality in Persons With Hidradenitis Suppurativa: Factors Associated With Sexual Desire and Functioning Impairment. Front. Psychiatry.

[33] Cuenca-Barrales C., Molina-Leyva A. (2020). Sexuality in Patients with Hidradenitis Suppurativa: Beliefs, Behaviors and Needs. Int. J. Environ. Res. Public Health.

[34] Yee D., Collier E.K., Atluri S., Jaros J., Shi V.Y., Hsiao J.L. (2020). Gender differences in sexual health impairment in hidradenitis suppurativa: A systematic review. Int. J. Womens Dermatol..

[35] Cuenca-Barrales C., Montero-Vílchez T., Szepietowski J., Matusiak L., Molina-Leyva A. (2021). Sexual impairment in patients with hidradenitis suppurativa: A systematic review. J. Eur. Acad. Dermatol. Venereol..

[36] Rosen R.C. (2000). Prevalence and risk factors of sexual dysfunction in men and women. Curr. Psychiatry Rep..

[37] Seetan K., Al-Zubi M., Al-Omari R. (2021). Sexual Dysfunction in Patients with Hidradenitis Suppurativa: A Systematic Review and Meta-Analysis. J. Clin. Aesthetic Dermatol..

[38] Kędra K., Janeczko K., Michalik I., Reich A. (2022). Sexual Dysfunction in Women and Men with Psoriasis: A Cross-Sectional Questionnaire-Based Study. Medicina.

[39] Horváth B., Janse I.C., Blok J.L., Driessen R.J., Boer J., Mekkes J.R., Prens E.P., van der Zee H.H. (2017). Hurley Staging Refined: A Proposal by the Dutch Hidradenitis Suppurativa Expert Group. Acta Derm. Venereol..

[40] Bhosle M.J., Kulkarni A., Feldman S.R., Balkrishnan R. (2006). Quality of life in patients with psoriasis. Health Qual. Life Outcomes.

[41] Misery L., Finlay A.Y., Martin N., Boussetta S., Nguyen C., Myon E., Taieb C. (2007). Atopic dermatitis: Impact on the quality of life of patients and their partners. Dermatology.

